# Multi-Site Musculoskeletal Symptoms in the Electronics Manufacturing Industry in China: A Cross-Sectional Study

**DOI:** 10.3390/ijerph192013315

**Published:** 2022-10-15

**Authors:** Yan Yin, Niu Di, Weiwei Guo, Wenbin Ding, Ning Jia, Zhongxu Wang, Feng Yang

**Affiliations:** 1 Department of Occupational Health and Poisoning Control, Division of Health Risk Factors Monitoring and Control, Shanghai Municipal Center for Disease Control and Prevention, Shanghai 200336, China; 2 National Institute of Occupational Health and Poison Control, Chinese Center for Disease Control and Prevention, Beijing 100050, China

**Keywords:** work-related musculoskeletal disorders, multi-site, electronics manufacturing industry, epidemiology

## Abstract

Background: With the development of the electronics manufacturing industry, the demand for human resources has increased, which has also led to the frequent occurrence of multi-site work-related musculoskeletal disorders. Method: The participants (*n* = 7307) were recruited from 30 enterprises in China in 2018. The prevalence of musculoskeletal disorders was estimated using a modified Chinese version of the Musculoskeletal Disorders Questionnaire. The multivariate logistic regression model was applied to evaluate the effects of risk factors on multi-site musculoskeletal symptoms. Additionally, the log-binomial model was established to examine the correlation between the various sites of musculoskeletal symptoms in the body. Results: The 12-month prevalence of musculoskeletal symptoms among participants was 40.6%. The proportion of musculoskeletal symptoms involving single-site and 2/>2 body sites were 11.6% and 29.0%, respectively. The results of logistic regression showed that female adults who smoked, had >5-year job tenure, and always stood or sat for long period at work had a higher risk in 2/>2 body sites of musculoskeletal symptoms (*p* < 0.05). However, physical exercise during leisure time and “Squatting or kneeling for long period at work” were more frequently protective factors. Furthermore, the log-binomial model indicated that the neck and shoulder were significantly related to each other (Prevalence Ratio, PR: 5.511 and 7.017). Conclusions: Several demographic characteristics and work-related factors were associated with multi-site musculoskeletal symptoms in the electronics manufacturing industry in China. Improving the levels of physical exercise and reducing posture problems and force loads may help to promote the health of workers.

## 1. Introduction

Musculoskeletal disorders, which exhibited a 30.7% increase in age-standardized disability-adjusted life years (DALYs) between 1990 and 2019, are one of the ten most important drivers of increasing burden [1]. Due to their association with few deaths, despite being large contributors to disability, musculoskeletal conditions have largely been ignored by the public. However, musculoskeletal disorders have become an increasingly large component of disease burden and, importantly, a larger component of health expenditure [1]. In China, the prevalence of musculoskeletal disorders has shown a trend of rapid growth in recent years, especially that of work-related disorders.

The electronics industry in China has experienced explosive growth since the mid-1990s, leading to a greater demand for labor [2]. As a result of the high-speed, single, repetitive characteristics of electronics manufacturing, workers are frequently forced to work in a certain posture [3]. To maintain this particular posture requires mobilizing two or more body sites. Previous studies indicated that the prevalence of musculoskeletal disorders in multiple body sites was higher than that in a single site [4,5]. There were several multi-site occurrence patterns of musculoskeletal symptoms in different positions, such as a “neck–shoulder–upper back–lower back” pattern among manufacturing workers [6]. Many studies have reported that personal, socioeconomic, and operational factors are associated with musculoskeletal symptoms in different body sites. A cross-sectional study based on 931 workers in Taiwan demonstrated that workers of an older age, that undertook repetitive and overburdened tasks, were at great risk of serious illness [7]. A longitudinal study carried out in a trailer assembly factory showed that a high work load may induce a higher rate of sick leave and upper limb musculoskeletal disorders (Hazard Ratio: 3.2, *p* < 0.05) [8], with the rate being higher in women than men.

At present, few studies have been focused on musculoskeletal symptoms, particularly multi-site musculoskeletal symptoms, in the electronics manufacturing industry, and have mainly been conducted in the Middle East, South Asia, and Southeast Asia [9,10,11,12]. The correlations of the incidence of musculoskeletal symptoms in different body sites are frequently ignored, which may cause several complications. Additionally, better treatment of these relationships would alleviate pain and prevent the progression of some relevant diseases. Moreover, most of the existing studies have few large national samples, and tend to only report regional results. Therefore, we conducted this study to examine associations between the prevalence of multi-site musculoskeletal symptoms, demographic characteristics, and work-related factors of the electronics manufacturing industry in China. Meanwhile, this study also explored the connections between musculoskeletal symptoms in different body sites.

## 2. Material and Methods

### 2.1. Study Population 

Study participants were from 30 electronics manufacturing enterprises (including semiconductor manufacturing, electronic chip manufacturing, computer manufacturing operations and so on) in North China, South China, East China and southwest of China between January and October in 2018, using a stratified cluster sampling method, only accepting participants with independent literacy skills. According to their size, all enterprises were classified as either large, medium, or small. All operators who were exposed to ergonomic factors were extracted for investigation. After standardization, this study contained 44 job titles in 40 kinds of workshops, including assembly, welding, packaging, testing, and wafer fabrication workers. These participants frequently sat or stood for a prolonged time, made repetitive movements, or worked in an uncomfortable position. Overall, 8036 participants responded to the invitations. Participants were excluded if they had a congenital spinal deformity or musculoskeletal disorders caused by non-work factors such as trauma, infectious diseases, and malignant tumors, and who had worked in the position for less than 1 year. As a consequence, a total of 7307 workers were included. The study received ethical approval from the Scientific Review and Ethics Review Committee of the Chinese Center for Disease Control and Prevention. Additionally, written informed consent was obtained from each participant at their enrollment.

### 2.2. Data Collection

A modified Chinese version of the Musculoskeletal Disorders Questionnaire, which integrated the contents of the general standardized Nordic Musculoskeletal Questionnaire (NMQ) and Dutch Musculoskeletal Questionnaire (DMQ), was used to conduct an epidemiological cross-sectional investigation of musculoskeletal symptoms [13,14]. In China, the scale was a feasible tool for assessing musculoskeletal symptoms and had good reliability and validity [15,16]. In this study, cases are defined according to the criteria from the National Institute for Occupational Safety and Health (NIOSH): (1) discomfort within the past year; (2) discomfort beginning after employment in the current job; (3) no prior accident or sudden injury (affecting a focal area of discomfort); and (4) episodes of discomfort occurring monthly or, if not every month, at least exceeding a week-long period of discomfort. 

The questionnaire contains 3 parts: demographic characteristics, musculoskeletal symptoms, and work-related factors. The first part involves gender, age, education level, marital status, job tenure, and others. The second part describes musculoskeletal symptoms (discomfort occurring every month or lasting for more than 1 week) in the past 12 months in 9 body regions: neck, shoulder, upper back, lower back, elbow, wrist/hand, hip/thigh, knee, and ankle/foot. Finally, work-related factors included job demand, working time, and break and work posture. Additionally, job demand was considered in this article, including standing for a long period at work, sitting for long period at work, squatting or kneeling for long period at work, working in an uncomfortable position, performing repetitive operations per minute, carrying heavy objects >5 kg, exerting great force with arms or hands, using vibrating tools at work, and working in cold or temperature-changing environments. In order to control the quality of the prevalence of musculoskeletal symptoms, the investigators were trained uniformly, and the subjects filled in the questionnaire centrally to ensure authenticity, integrity, and a high recovery rate.

### 2.3. Statistical Analysis

Descriptive analyses were conducted for demographic and occupational characteristics including gender (“Male” and “Female”), age (“<26 years”, “26–35 years”, and “>35 years”), body mass index (BMI, “<18.5 kg/m^2^”, “18.5–23.9 kg/m^2^”, “24–27.9 kg/m^2^”, and “≥28 kg/m^2^”), education level (“Junior middle school or below”, “Senior high school or technical secondary school”, and “Bachelor degree or above”), marital status (“Single”, “Married”, and “Divorced”), monthly income (“≤3000 RMB”, “3001–5000 RMB”, and “>5000 RMB”), physical exercise (“Never”, “Occasionally”, “2–3 times/month”, “1–2 times/week”, and “More than 3 times/week”), smoking behavior (“Never”, “Occasionally”, and “Frequently”), job tenure (“1–2 years”, “3–5 years”, and “>5 years”), and more than 9 work-related factors (“Never”, “Occasionally”, “Frequently”, and “Always”), as shown in Table 1. Differences in the distribution of these factors between groups were tested using the Chi-square test. Furthermore, we summarized the positive cases and positive rate of multi-site musculoskeletal symptoms. The multivariate logistic regression models were used to examine the influence of potential risk factors on the occurrence of musculoskeletal symptoms in the past 12 months. The lowest-level group was taken as the reference group, and the other three groups were set as dummy variables to be input into the model for the trend test. According to the results of the Chi-square test, the factors that had significant differences between groups were included in the models. All the results are shown as an odds ratio (OR) with a 95% confidence interval (95%CI). In order to evaluate the correlation between various body sites of musculoskeletal symptoms, the log-binomial models were employed to calculate the prevalence ratio (PR) [17,18]. Finally, according to the PR, we described the prevalence of concurrent musculoskeletal symptoms in two body sites.

All statistical analyses were performed using R software (version 3.6.2, R Core Team, Vienna, Austria). The “logbin” packages were used for the log-binomial model. *p* < 0.05 indicates statistical significance.

## 3. Results

A summary of the participants’ basic information is shown in Table 1. Men (45.6%) and women (54.4%) were evenly distributed among participants. The majority were young, with a mean (SD) age of 32.36 (7.39) years. Their average level of BMI was 22.79 (4.75) kg/m^2^. Moreover, most of the participants were less educated, with an education level of senior high school or below (75.5%). Of all participants, 5636 (77.1%) never smoke and 4272 (58.5%) exercise occasionally. Those that had been working in their current position for less than 5 years accounted for 70.4%. We found that most of the participants worked in a standing or sitting posture (standing: 68.7%; sitting: 75.7%), rarely squatting or kneeling (24.9%). A total of 72.9% of the participants exerted great force with their arms or hands and 75.1% performed repetitive operations per minute.

The positive cases and positive rate of multi-site musculoskeletal symptoms are shown in Table 2. In total, 2964 cases of musculoskeletal symptoms were identified with a prevalence of 40.6%. The results indicate that the prevalence of multiple sites was higher than that of a single site, with the highest prevalence in two body sites. The positive rate of musculoskeletal symptoms in two body sites was 8.8%, accounting for 21.7% of the overall prevalence. Few participants had concurrent musculoskeletal symptoms in more than 6 body sites (3.5%), accounting for 8.6% of the overall prevalence.

According to the results of Table 1 and Table 2, we performed a Chi-square test for the prevalence of two sites of musculoskeletal symptoms according to different demographic and work-related factors, as shown in the Supplementary Material (Table S1). Among all demographic characteristics, we observed that the prevalence of musculoskeletal symptoms in women was significantly higher than that in men (*p* < 0.01). The prevalence was significantly higher in participants who performed less physical exercise than in those who exercised regularly (*p* < 0.05). Additionally, the prevalence was higher in workers with longer job tenure than those with shorter job tenure (*p* < 0.05). The results show significant differences between groups with musculoskeletal symptoms and those without in terms of almost all work-related factors. Therefore, age, gender, marital status, physical exercise, monthly income (RMB), job tenure (years), and work-related risk factors with significant differences were included in the multivariate logistic regression analyses. In accordance with previous studies, we considered smoking behavior and other potential factors in the analyses [6,19,20,21].

**Figure 1:**
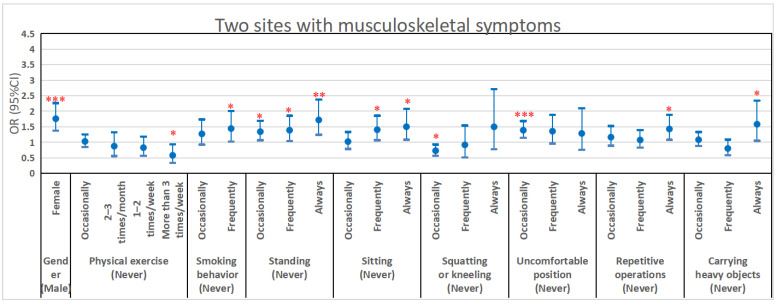
Multivariate logistic regression analysis describing the associations between demographic and work-related factors with two body sites musculoskeletal symptoms. Notes: *, *p* < 0.05; **, *p* < 0.01; ***, *p* < 0.001. Models consisted of age, gender, monthly income (RMB), marital status, physical exercise, smoking status, job tenure (years), standing for long period at work, sitting for long period at work, squatting or kneeling for long period at work, working in an uncomfortable position, performing the repetitive operations per minute, carrying heavy objects >5 Kg, exerting great force with arms or hands, use vibrating tools at work and working in cold or temperature changing environments.

The results of multivariate logistic regression analyses are shown in Figure 1 and Figure S1, Tables S1 and S2. Because of the large amount of data, the picture results only show the factors that were significantly varied. We observed that gender, physical exercise, smoking behavior, and six work-related factors had a significant impact on the risk of concurrent musculoskeletal symptoms in two body sites, as shown in Figure 1. The risk in women was higher than that in men (OR [95%CI]: 1.760 [1.377, 2.263], *p* < 0.001). The risk of concurrent musculoskeletal symptoms in two body sites also increased with frequent smoking behavior (OR [95%CI]: 1.443 [1.028, 2.009], *p* value for trend = 0.001). However, physical exercise was a protective factor. A greater level of physical exercise (more than 3 times/week) reduced the risk of musculoskeletal symptoms in two body sites (OR [95%CI]: 0.581 [0.342, 0.934], *p* value for trend = 0.037). Workers in the electronics manufacturing industry who stood for long periods at work, sat for long periods at work, performed repetitive operations per minute, and carried heavy objects were more prone to musculoskeletal symptoms (OR [95%CI]: 1.721 [1.243, 2.375], *p* value for trend = 0.005; 1.502 [1.082, 2.083], *p* value for trend = 0.005; 1.429 [1.082, 1.890], *p* value for trend = 0.022 and 1.583 [1.053, 2.343], *p* value for trend = 0.445). However, participants that occasionally squatted or kneeled for long period at work had a lower risk than those who never did so (OR [95%CI]: 0.730 [0.570, 0.929], *p* value for trend = 0.301). In addition to the factors mentioned above, age, monthly income, job tenure, and “exerting great force with arms or hands” had a significant influence on the risk of concurrent musculoskeletal symptoms in more than two body sites. Details are shown in Figure S1 and Table S2.

In Table 3 are shown the correlations between musculoskeletal symptoms of different body sites. The horizontal target is the independent variable, and the vertical target is the dependent variable. We observed that musculoskeletal symptoms in the shoulder, upper back, and lower back were associated with musculoskeletal symptoms in the neck (PR: 5.511, 1.223, and 1.154, *p* < 0.001). Additionally, musculoskeletal symptoms in the neck, upper back, and lower back were associated with musculoskeletal symptoms in the shoulder (PR: 7.017, 1.668, and 1.098, *p* < 0.01). Moreover, the PRs of musculoskeletal symptoms in the hip/thigh and knee leading to musculoskeletal symptoms in the ankle/foot were 2.922 and 2.385, respectively. In summary, musculoskeletal symptoms in the neck, shoulder, upper back, and lower back were associated with each other. Moreover, musculoskeletal symptoms in the hip/thigh, knee, and ankle/foot were also related to each other.

Musculoskeletal symptoms in two body sites had the highest prevalence; the prevalence of two sites exhibiting concurrent musculoskeletal symptoms is summarized in Table 4. The results indicate that “neck and shoulder” were major sites of concurrent musculoskeletal symptoms in two body sites, and the number of positive cases (positive rate) was 246 (3.4%), accounting for 38.2% of the total number of positive cases. Musculoskeletal symptoms in the “neck and lower back”, “shoulder and upper back”, and “neck and upper back” ranked second, third, and fourth in terms of prevalence, respectively. However, very few participants suffered from musculoskeletal symptoms in the lower limbs. Evidence was also provided by the prevalence of single-site musculoskeletal symptoms (Table 5). The neck, shoulder, upper back, and lower back were prone to musculoskeletal symptoms (positive rate of a single site: 4.2%, 1.7%, 1.5%, and 0.8%).

In addition, we observed that gender, job tenure, and sitting for a long period at work had a significant impact on the risk of concurrent musculoskeletal symptoms in the neck and shoulder (Figure S2). The results show that greater job tenure (more than 5 years) increased the risk of musculoskeletal symptoms in the neck and shoulder (OR [95%CI]: 1.456 [1.032, 2.059], *p* < 0.05). Women and workers who frequently sat for long periods at work were more prone to musculoskeletal symptoms in the neck and shoulder.

## 4. Discussion

Our study reveals that most of the workers in the electronics manufacturing industry perform repetitive tasks in a standing or sitting position, and factors such as gender, physical exercise, job tenure, and sitting for long period at work have an impact on multi-site musculoskeletal symptoms. Musculoskeletal symptoms in the neck and shoulder were most closely associated and musculoskeletal symptoms in these two sites had the highest prevalence.

To date, epidemiological evidence is limited in China, and even globally, with regard to the study of multi-site musculoskeletal symptoms in the electronics manufacturing industry, focusing on certain body sites [9,10,11,19]. The total 12-month prevalence of musculoskeletal symptoms among the study subjects was 40.6%. The prevalence of symptoms in the neck and shoulder was 26.8% and 22.8%, respectively. A cross-section study conducted in the thin-film transistor-liquid crystal display manufacturing industry reported that the workers frequently developed musculoskeletal symptoms in the shoulder (59.8%), neck (49.5%), and upper back (30.6%) [22]. Previous studies among female workers in the electronics industry showed that the neck and shoulder were the most vulnerable to musculoskeletal disorders. After reassignment to more varied tasks, the musculoskeletal symptoms of some participants improved [23,24]. Consistent with these studies, our study also indicates that the neck and shoulder symptoms are the most common musculoskeletal disorders in the electronics industry. However, a cross-sectional study in Malaysia reported that the prevalence of female workers in the semiconductor industry was up to 83.4%, and the most common body sites of musculoskeletal symptoms were the back (57.8%), lower leg (48.4%), and shoulder (44.8%) [19]. Another study conducted in Peninsular Malaysia including 906 participants found similar results, and reported the prevalence of musculoskeletal symptoms to be 80.50% [12]. The prevalence of musculoskeletal disorders was far beyond the results of our study. Through assessing the results of existing articles, we believe that controls for gender and the specific type of work were sources of variance. The above studies mainly investigated female workers and focused on certain assembly lines, and many studies have reported that women are more likely to develop musculoskeletal symptoms [18,21].

The results of multivariate logistic regression analyses also show that women are more affected by multi-site musculoskeletal symptoms. This suggests that female workers are more likely to suffer from multi-site musculoskeletal symptoms, which may be related to the weaker body load capacity of women compared with men. The sex differences in musculoskeletal symptoms also existed between different job categories, which resulted from the different exposures to repetitive biomechanical constraints at work [25]. Men in the factory had a higher opportunity of exposure to force than women. In our study, 60.83% men undertook the task of carrying heavy objects, whereas fewer women (22.12%) were assigned to such tasks. We found that women represented a higher proportion of assembly, welding, packaging, and testing workers (χ^2^ [*p* value]: 13.718 [<0.001], 6.338 [0.012], 5.560 [0.018] and 8.563 [0.003]). The jobs in these workshops require holding a fixed posture and performing repetitive operations for a long time, such that the muscle group of the neck and shoulder cannot relax and becomes tense and stiff. However, women reported a larger hardness of the shoulder muscle compared with men [26], putting them at increased risk of developing musculoskeletal disorders in these job titles. Female adults almost represent the main labor force in electronics manufacturing assembly lines. The global age-standardized DALY rate of musculoskeletal disorders was higher in women than in men across age groups during the period of 1990–2019 [27]. Therefore, musculoskeletal symptoms in women must be taken seriously. The risk of multi-site musculoskeletal symptoms was reduced by more frequent physical exercise. An article by Ribas et al., along with other reports, highlighted the essential effects of physical exercise outside of the workplace, especially when performed in a more intense and systematic manner. Physical exercise could be an important factor in the protection of workers’ functional capacity and the prevention of musculoskeletal symptoms and pain [28]. Some of these studies also reported the positive benefits of strength training programs in improving overall muscle strength in the neck–shoulder region [29,30]. We found there was no significant association between job tenure and the presence of two sites with musculoskeletal symptoms in the past 12 months, which is inconsistent with previous studies about the prevalence of single-site musculoskeletal symptoms [8,19]. However, the associations between job tenure and more than two sites of musculoskeletal disorders were significant. It is likely that, due to the fact that most workers have worked for only a short period of time in their current position (<5 years: 70.4%), the effects of exposure have not yet clearly appeared. 

In the models, the work-related factors included strength load and postural load. We observed that staying in a standing position for long periods of time increased the risk of musculoskeletal symptoms. In order to maintain a standing position, the lower extremities, hips, and spine require constant muscle contractions. This condition induces pressure on the ligaments and the spine, and the discs tend to hit the nerves, causing pain [31]. As the time and frequency of assuming a sitting position increased, the risk of musculoskeletal disorders also increased. The results of trend tests with regard to standing or sitting for long periods suggest that damage to the musculoskeletal system may be cumulative [6]. However, the effect of other risk factors on musculoskeletal symptoms did not show a significant trend, such as squatting or kneeling for long periods at work. Table 1 shows that the subjects in the electronics manufacturing industry mainly remained in a standing or sitting position for long periods, rather than squatting or kneeling. Therefore, occasionally squatting or kneeling at work may relieve musculoskeletal fatigue caused by standing or sitting for a long time. In addition, carrying heavy objects and exerting great force with the arms or hands are able to increase muscle fatigue, which causes irreversible damage. 

On the basis of the relevant literature, we have fully considered the correlation between musculoskeletal symptoms in different body sites [6]. We found that musculoskeletal symptoms in the neck, shoulder, upper back, and lower back were interrelated, which was in line with the occurrence pattern of musculoskeletal symptoms in the multiple sites of “neck, shoulder, upper back, and lower back” proposed by previous studies [6,32]. However, their biological mechanisms are complex and are not yet well understood. A plausible pathway to explain the correlation and the pain is that ischemia and hypoxia lead to an energy crisis for muscles or tendons that sustain high static loads [33,34]. A previous publication indicated a role of local muscular processes, being causally related to this association [35]. In addition, we also observed a correlation between musculoskeletal symptoms in the hip/thigh, knee, and ankle/foot. This correlation is likely to be associated with the physiological structure of the human body. At present, no studies have proposed this occurrence pattern of musculoskeletal symptoms in workers in the electronics manufacturing industry. 

Synthesizing the results of our study, the following suggestions are put forward for the construction of the electronic manufacturing industry. To avoid workers staying in an uncomfortable position, employers could set up adjustable workstations, such as sit–stand workstations [36,37]. Employees could independently decide the height of the working platform and working posture. In fact, due to the limitation of the work category and content, many working tasks must be completed in a sitting position. Employers should provide workers with adjustable chairs which can support the back and legs, so as to reduce the muscle fatigue in the neck, shoulder, and back caused by long-term operation in the sitting position. Special tools for work should be provided if these changes cannot be made, such as a self-created tilted work platform for assembly line workers [9]. In order to relieve muscle fatigue throughout the body, workers could stretch and rest at regular intervals, prompted by broadcast [38,39]. Due to their different bodily anatomy, women are subjected to greater ergonomic stress than men. Employers should pay more attention to the health of female workers, allowing them to work in a suitable position and providing the complete personal protective equipment. To fundamentally solve the occurrence of musculoskeletal disorders, it is necessary to rationally optimize the process flow, which could reduce the number of repetitive operations in assemblies and other work. Finally, the workers’ awareness of prevention methods for musculoskeletal disorders could be improved through enhanced educational training.

The sample size of our study is large and representative, but the study also has several limitations. First, due to the cross-sectional design and the unavailability of the exact onset date of musculoskeletal symptoms, the findings of this study cannot confirm the causal relationship between risk factors and musculoskeletal symptoms. Further longitudinal studies are needed. Second, we used a questionnaire to screen the musculoskeletal symptoms, a subjective method without clinical diagnoses, which may have led to diagnostic errors. The exposure to work-related risk factors was also filled in according to personal understanding. In addition, posture (having to hold joints in a certain position for extended (time defined) periods, twisting and rotating, and reaching overhead) and force (grasping or pinching, or shocks from tool or equipment) were also associated with musculoskeletal symptoms in previous studies, but we were not able to consider them in this study due to the unavailability of data on the measurement of other work-related risk factors. We will collect and analyze relevant data in subsequent cohort and intervention studies. Finally, we did not consider the impact of psychological and management factors on multi-site musculoskeletal symptoms, which could be associated with a certain degree of pain and stress.

## 5. Conclusions

Similar to previous epidemiologic studies, the results of our logistic regression indicate that demographic characteristics and work-related factors are associated with multi-site musculoskeletal symptoms. According to these findings, more frequent physical exercise, appropriate rest, and reductions in strength and postural load may help to prevent musculoskeletal symptoms, reduce economic burden, and optimize the healthy labor force. Policy makers should pay more attention to the musculoskeletal symptoms of workers and consider them as legal occupational diseases at an earlier date. The government should put in place effective policies and measures to protect workers in all kinds of industries from musculoskeletal symptoms.

## Figures and Tables

**Table 1:** Basic characteristics of study participants.

Variables	*n*	(%)	χ^2^	*p* Value
**Gender**			133.880	<0.001
Male	3334	45.6		
Female	3973	54.4		
**Age (years old)**			29.964	<0.001
<26	1344	18.4		
26–35	3800	52.0		
>35	2163	29.6		
**BMI (kg/m^2^, NA = 65)**			16.731	0.002
<18.5	678	9.3		
18.5–23.9	4616	63.2		
24–27.9	1412	19.3		
≥28	536	7.3		
**Education level**			6.640	0.084
Junior middle school or below	1960	26.8		
Senior high school or technical secondary school	3558	48.7		
Junior college	1118	15.3		
Bachelor degree or above	671	9.2		
**Marital status**			38.772	<0.001
Single	2324	31.8		
Married	4840	66.2		
Divorced	143	2.0		
**Monthly income (RMB)**			171.070	<0.001
≤3000	1517	20.8		
3001–5000	4144	56.7		
>5000	1646	22.5		
**Physical exercise**			54.342	<0.001
Never	1759	24.1		
Occasionally	4272	58.5		
2–3 times/month	343	4.7		
1–2 times/week	566	7.7		
More than 3 times/week	367	5.0		
**Smoking behavior**			54.213	<0.001
Never	5716	78.2		
Occasionally	919	12.6		
Frequently	672	9.2		
**Job tenure (years)**			74.593	<0.001
1–2	3335	45.6		
3–5	1815	24.8		
>5	2157	29.5		
**Standing for long period at work**			81.039	<0.001
Never	2285	31.3		
Occasionally	2385	32.6		
Frequently	1619	22.2		
Always	1018	13.9		
**Sitting for long period at work**			193.120	<0.001
Never	1779	24.3		
Occasionally	2177	29.8		
Frequently	2310	31.6		
Always	1041	14.2		
**Squatting or kneeling for long period at work**			20.069	<0.001
Never	5486	75.1		
Occasionally	1542	21.1		
Frequently	189	2.6		
Always	90	1.2		
**Work in an uncomfortable position**			398.610	<0.001
Never	4269	58.4		
Occasionally	2371	32.4		
Frequently	478	6.5		
Always	189	2.6		
**Performing the repetitive operations per minute**			524.770	<0.001
Never	1823	24.9		
Occasionally	1765	24.2		
Frequently	2047	28.0		
Always	1672	22.9		
**Carrying heavy objects** > 5 Kg			100.780	<0.001
Never	3395	46.5		
Occasionally	2591	35.5		
Frequently	963	13.2		
Always	358	4.9		
**Exerting great force with arms or hands**			282.860	<0.001
Never	1977	27.1		
Occasionally	2241	30.7		
Frequently	1928	26.4		
Always	1161	15.9		
**Using vibrating tools at work**			38.941	<0.001
Never	5772	79.0		
Occasionally	1119	15.3		
Frequently	239	3.3		
Always	177	2.4		
**Working in cold or temperature changing environments**			15.545	<0.001
Yes	1361	18.6		
No	5964	81.4		
**Total**	7307	100.0		

**Table 2:** Prevalence of multi-site musculoskeletal symptoms in the past 12 months.

Body Sites with Musculoskeletal Symptoms	Positive Case	Positive Rate	Positive Proportion
0	4343	59.4	0
1	848	11.6	28.6
2	644	8.8	21.7
3	508	7.0	17.1
4	361	4.9	12.2
5	212	2.9	7.2
6	136	1.9	4.6
>6	255	3.5	8.6
Total	7307	100.0	100.0

**Table 3:** PR value of the musculoskeletal symptoms of different body sites in the past 12 months. (*n* = 2116).

Affected Sits of Musculoskeletal Symptoms	Affected Sits of Musculoskeletal Symptoms								
	Neck	Shoulder	Upper Back	Lower Back	Elbow	Wrist/Hand	Hip/Thigh	Knee	Ankle/Foot
Neck		7.017 ***	2.381 ***	2.589 ***	0.989	1.977 ***	1.398 ***	1.098	1.737 ***
Shoulder	5.511 ***		4.232 ***	1.619 ***	1.895 ***	2.622 ***	1.421 ***	1.467 ***	1.013
Upper back	1.223 ***	1.668 ***		2.115 ***	1.559 ***	1.092	1.582 ***	1.111	0.960
Lower back	1.154 ***	1.098 **	1.801 ***		1.442 ***	1.219 **	1.491 ***	1.574 ***	1.148
Elbow	0.954 *	1.026	1.110 *	1.115		2.603 ***	1.120	1.424 ***	0.980
Wrist/Hand	1.070 **	1.093 **	1.088	1.187 **	5.782 ***		1.397 ***	1.345 **	1.481 ***
Hip/Thigh	1.030	1.013	1.270 ***	1.277 ***	1.660 ***	1.242 **		2.418 ***	2.922 ***
Knee	0.991	1.035	1.012	1.198 **	1.471 ***	1.046	1.612 ***		2.385 ***
Ankle/Foot	1.030	0.987	0.993	1.061	1.052	1.195 **	2.601 ***	3.019 ***	

Notes: *, *p* < 0.05; **, *p* < 0.01; ***, *p* < 0.001.

**Table 4:** Distribution of concurrent musculoskeletal symptoms in two body sites of participants in the past 12 months. (*n* = 644).

Body Sites with Musculoskeletal Symptoms	Positive Case	Positive Rate (%)	Positive Proportion (%)
Neck and shoulder	246	3.4	38.2
Neck and upper back	30	0.4	4.7
Neck and lower back	62	0.8	9.6
Neck and wrist/hand	24	0.3	3.7
Shoulder and upper back	34	0.5	5.3
Shoulder and lower back	17	0.2	2.6
Shoulder and wrist/hand	19	0.3	3.0
Upper back and lower back	15	0.2	2.3
Lower back and hip/thigh	14	0.2	2.2
Lower back and ankle/foot	14	0.2	2.2
Hip/Thigh and ankle/foot	16	0.2	2.5
others	153	2.1	23.8

**Table 5:** Prevalence of only single-site musculoskeletal symptom in the past 12 month. (*n* = 848).

Body Sites with Musculoskeletal Symptoms	Positive Case	Positive Rate (%)	Positive Proportion (%)
Neck	304	4.2	35.8
Shoulder	124	1.7	14.6
Upper back	55	0.8	6.5
Lower back	109	1.5	12.9
Elbow	23	0.3	2.7
Wrist/Hand	48	0.7	5.7
Hip/Thigh	42	0.6	5.0
Knee	41	0.6	4.8
Ankle/Foot	102	1.4	12.0

## Data Availability

The datasets used in the current study are available from the corresponding author on reasonable request.

## Supplementary Materials

The following supporting information can be downloaded at: https://www.mdpi.com/article/10.3390/ijerph192013315/s1, Figure S1: Multivariate logistic regression analysis describing the associations between demographic and work-related factors with more than two body sites musculoskeletal symptoms; Figure S2: Multivariate logistic regression analysis describing the associations between demographic and work-related factors of the neck and shoulder musculoskeletal symptoms; Table S1: Chi-square test for different demographic characteristics and work-related factors; Table S2: Multivariate logistic regression analysis describing the associations between demographic and work-related factors with two body sites musculoskeletal symptoms; Table S3: Multivariate logistic regression analysis describing the associations between demographic and work-related factors with more than two body sites musculoskeletal symptoms.

## References

[1] Collaborators GDaI (2020). Global burden of 369 diseases and injuries in 204 countries and territories, 1990–2019: A systematic analysis for the Global Burden of Disease Study 2019. Lancet.

[2] Zhao L. (2018). Research on the development of China’s electric manufacturing Industry. Mod. Ind. Econ. Inf..

[3] Ma Y.Y., Liu K.P., Ruan Y.M., Liu Y.M., Yang Y., Peng Z.H., Jia N., Wang Z.X. (2019). Research progress on work-related musculoskeletal disorders in electronics manufacturing workers. Chin. J. Ind. Med..

[4] Fumei K., Yongle S., Bin F., Zhongxu W. (2021). An investigation of musculoskeletal disorders at multiple sites and related influencing factors among workers in an automobile assembly shop. Chin. J. Ind. Hyg. Occup. Dis..

[5] Zhiheng P., Peixian C., Yan Y., Hai Z., Yimin L., Shaoxue H., Qiangbing Y., Ning J., Zhongxu W., Zhi W. (2021). Analysis risks of muti-site work-related musculoskeletal disorders of painters in manufacturing industry. Occup. Health Emerg. Rescue.

[6] Wang F.J., Jin X., Nazakat M., Dong Y.D., Wang S.J., Zhang Z.B., Yu S.F., Yang L.Y., He L.H. (2020). Occurrence pattern of musculoskeletal disorders and its influencing factors among manufacturing workers. Beijing Da Xue Xue Bao. Yi Xue Ban = J. Peking University. Health Sci..

[7] Chu P.C., Wang T.G., Guo Y.L. (2021). Work-related and personal factors in shoulder disorders among electronics workers: Findings from an electronics enterprise in Taiwan. BMC Public Health.

[8] Häkkänen M., Viikari-Juntura E., Martikainen R. (2001). Job experience, work load, and risk of musculoskeletal disorders. Occup. Environ. Med..

[9] Daneshmandi H., Kee D., Kamalinia M., Oliaei M., Mohammadi H. (2018). An ergonomic intervention to relieve musculoskeletal symptoms of assembly line workers at an electronic parts manufacturer in Iran. Work (Read. Mass.).

[10] Aghilinejad M., Azar N.S., Ghasemi M.S., Dehghan N., Mokamelkhah E.K. (2016). An ergonomic intervention to reduce musculoskeletal discomfort among semiconductor assembly workers. Work (Read. Mass.).

[11] Chee H.L., Rampal K.G. (2004). Work-related musculoskeletal problems among women workers in the semiconductor industry in Peninsular Malaysia. Int. J. Occup. Environ. Health.

[12] Chee H.L., Rampal K.G., Chandrasakaran A. (2004). Ergonomic risk factors of work processes in the semiconductor industry in Peninsular Malaysia. Ind. Health.

[13] Kuorinka I., Jonsson B., Kilbom A., Vinterberg H., Biering-Sørensen F., Andersson G., Jørgensen K. (1987). Standardised Nordic questionnaires for the analysis of musculoskeletal symptoms. Appl. Ergon..

[14] Hildebrandt V.H., Bongers P.M., van Dijk F.J., Kemper H.C., Dul J. (2001). Dutch Musculoskeletal Questionnaire: Description and basic qualities. Ergonomics.

[15] Dong Y.D., Nazakat M., Wang F.J., Jin X., Wang S., He L.H., Yu S.F., Zhang Z.B., Wang Y., Sheng L.G. (2019). Establishment and verification of the Chinese Musculoskeletal Questionnaire—The questionnaire is attached in the attachment. China Occup. Med..

[16] Yang L., Hildebrandt V.H., Yu S.F., Ling R.J., He L.H., Chen W.H., Xia Z.L., Wang J.X., Li L.P., Wang S. (2009). Musculoskeletal Disorders Questionnaire Introduction Attached questionnaire. Ind. Health Occup. Dis..

[17] Donoghoe M.W., Marschner I.C. (2018). logbin: An R Package for Relative Risk Regression Using the Log-Binomial Model. J. Stat. Softw..

[18] Haukka E., Leino-Arjas P., Solovieva S., Ranta R., Viikari-Juntura E., Riihimäki H. (2006). Co-occurrence of musculoskeletal pain among female kitchen workers. Int. Arch. Occup. Environ. Health.

[19] Chandrasakaran A., Chee H.L., Rampal K.G., Tan G.L. (2003). The prevalence of musculoskeletal problems and risk factors among women assembly workers in the semiconductor industry. Med. J. Malays..

[20] Jia N., Zhang M., Zhang H., Ling R., Liu Y., Li G., Yin Y., Shao H., Zhang H., Qiu B. (2022). Prevalence and risk factors analysis for low back pain among occupational groups in key industries of China. BMC Public Health.

[21] Maimaiti N., Wang J., Jin X., Wang S., Qin D., He L., Wang F., Zhang Z., Forsman M., Yang L. (2019). Cervical musculoskeletal disorders and their relationships with personal and work-related factors among electronic assembly workers. J. Safety Res..

[22] Lu C.W., Yao C.C., Kuo C.W. (2021). The ergonomics approach for thin film transistor-liquid crystal display manufacturing process. Work (Read. Mass.).

[23] Kilbom Å., Persson J., Jonsson B.G. (1986). Disorders of the cervicobrachial region among female workers in the electronics industry. Int. J. Ind. Ergon..

[24] Jonsson B.G., Persson J., Kilbom Å. (1988). Disorders of the cervicobrachial region among female workers in the electronics industry: A two-year follow up. Int. J. Ind. Ergon..

[25] Silverstein B., Fan Z.J., Smith C.K., Bao S., Howard N., Spielholz P., Bonauto D., Viikari-Juntura E. (2009). Gender adjustment or stratification in discerning upper extremity musculoskeletal disorder risk?. Scand. J. Work. Environ. Health.

[26] Kimura T., Tsuda Y., Uchida S., Eboshida A. (2006). Association of perceived stress and stiff neck/shoulder with health status: Multiple regression models by gender. Hiroshima J. Med. Sci..

[27] Jin Z., Feng X., Wang D., Zhu Y., Liang J., Zhang H., Zhao J., Sun L. (2021). Global, regional, and national trends in sex- and age-specific disability adjusted life years of musculoskeletal disorders, 1990–2019. Rheumatology.

[28] Ribas T.M., Teodori R.M., Mescolotto F.F., Montebelo M.I.L., Baruki S.B.S., Pazzianotto-Forti E.M. (2021). Impact of physical activity levels on musculoskeletal symptoms and absenteeism of workers of a metallurgical company. Rev. Bras. De Med. Do Trab. Publicacao Of. Da Assoc. Nac. De Med. Do Trab.-ANAMT.

[29] Andersen L.L., Christensen K.B., Holtermann A., Poulsen O.M., Sjøgaard G., Pedersen M.T., Hansen E.A. (2010). Effect of physical exercise interventions on musculoskeletal pain in all body regions among office workers: A one-year randomized controlled trial. Man. Ther..

[30] Viljanen M., Malmivaara A., Uitti J., Rinne M., Palmroos P., Laippala P. (2003). Effectiveness of dynamic muscle training, relaxation training, or ordinary activity for chronic neck pain: Randomised controlled trial. BMJ (Clin. Res. Ed.).

[31] Nelson-Wong E., Gregory D.E., Winter D.A., Callaghan J.P. (2008). Gluteus medius muscle activation patterns as a predictor of low back pain during standing. Clin. Biomech..

[32] Beales D., Kyaw-Myint S., Smith A., OʼSullivan P., Pransky G., Linton S., Job J., Straker L. (2017). Work Productivity Loss in Young Workers Is Substantial and Is Associated With Spinal Pain and Mental Ill-health Conditions. J. Occup. Environ. Med..

[33] Sjøgaard G., Jensen B.R., Hargens A.R., Søgaard K. (2004). Intramuscular pressure and EMG relate during static contractions but dissociate with movement and fatigue. J. Appl. Physiol..

[34] Strøm V., Røe C., Knardahl S. (2009). Work-induced pain, trapezius blood flux, and muscle activity in workers with chronic shoulder and neck pain. Pain.

[35] Larsson B., Søgaard K., Rosendal L. (2007). Work related neck-shoulder pain: A review on magnitude, risk factors, biochemical characteristics, clinical picture and preventive interventions. Best Pract. Research. Clin. Rheumatol..

[36] Alkhajah T.A., Reeves M.M., Eakin E.G., Winkler E.A., Owen N., Healy G.N. (2012). Sit-stand workstations: A pilot intervention to reduce office sitting time. Am. J. Prev. Med..

[37] Danquah I.H., Kloster S., Holtermann A., Aadahl M., Tolstrup J.S. (2017). Effects on musculoskeletal pain from "Take a Stand!"—a cluster-randomized controlled trial reducing sitting time among office workers. Scand. J. Work. Environ. Health.

[38] Moore T.M. (1998). A workplace stretching program. Physiologic and perception measurements before and after participation. AAOHN J. Off. J. Am. Assoc. Occup. Health Nurses.

[39] Henning R.A., Jacques P., Kissel G.V., Sullivan A.B., Alteras-Webb S.M. (1997). Frequent short rest breaks from computer work: Effects on productivity and well-being at two field sites. Ergonomics.

