# Supplementary material for: Multi-Site Musculoskeletal Symptoms in the Electronics Manufacturing Industry in China: A Cross-Sectional Study
Source: Int J Environ Res Public Health. 2022 Oct 15;19(20):13315. doi: 10.3390/ijerph192013315 (PMC9602541; doi:10.3390/ijerph192013315)

Table S1. Chi-square test for different demographic characteristics and work-related factors.

Variables	Total			Two sites with WMSDs		
	Number of cases	χ^2	P value	Number of cases	χ^2	P value
**Gender**		133.880	<0.001		33.003	<0.001
Male	1,110			224		
Female	1,854			420		
**Age (years old)**		29.964	<0.001		8.337	0.015
<26	480			98		
26–35	1,515			328		
>35	969			218		
**BMI (kg/m^2, NA=65)**		16.731	0.002		7.797	0.099
<18.5	257			54		
18.5–23.9	1,850			411		
24–27.9	591			120		
≥28	249			58		
**Education level**		6.640	0.084		1.184	0.757
Junior middle school or below	786			172		
Senior high school or technical secondary school	1,489			304		
Junior college	421			103		
Bachelor degree or above	268			65		
**Marital status**		38.772	<0.001		6.683	0.035
Single	821			177		
Married	2,082			451		
Divorced	61			16		

**Monthly income (RMB)**		171.070	<0.001		6.937	0.031
≤3000	838			158		
3001–5000	1,519			339		
>5000	607			147		
**Physical exercise**		54.342	<0.001		9.725	0.045
Never	737			161		
Occasionally	1,798			396		
2–3 times/month	150			28		
1–2 times/week	183			40		
More than 3 times/week	96			19		
**Smoking behavior**		54.213	<0.001		3.044	0.218
Never	2,420			517		
Occasionally	271			67		
Frequently	273			60		
**Job tenure (years)**		74.593	<0.001		6.548	0.038
1–2	1,223			266		
3–5	703			163		
>5	1,038			215		
**Standing for long period at work**		81.039	<0.001		4.190	0.242
Never	982			206		
Occasionally	838			205		
Frequently	627			129		
Always	517			104		
**Sitting for long period at work**		193.120	<0.001		19.722	<0.001
Never	659			138		

Occasionally	724			160		
Frequently	976			228		
Always	605			118		
**Squatting or kneeling for long period at work**		20.069	<0.001		14.299	0.003
Never	2,222			509		
Occasionally	610			105		
Frequently	75			16		
Always	57			14		
**Work in an uncomfortable position**		398.610	<0.001		12.237	0.007
Never	1,365			335		
Occasionally	1,149			238		
Frequently	308			50		
Always	142			21		
**Performing the repetitive operations per minute**		524.770	<0.001		24.496	<0.001
Never	437			125		
Occasionally	598			144		
Frequently	923			183		
Always	1,006			192		
**Carrying heavy objects >5 Kg**		100.780	<0.001		9.663	0.022
Never	1,221			297		
Occasionally	1,075			233		
Frequently	456			69		
Always	212			45		
**Exerting great force with arms or hands**		282.860	<0.001		7.613	0.055
Never	644			178		

Occasionally	740			168		
Frequently	909			187		
Always	671			111		
**Use vibrating tools at work**		38.941	<0.001		7.334	0.062
Never	2,315			529		
Occasionally	427			78		
Frequently	114			25		
Always	108			12		
**Working in cold or temperature changing environments**		15.545	<0.001		0.225	1.473
Yes	617			108		
No	2,347			536		
**Total**	2,964			644		

Table S2 Multivariate logistic regression analysis describing the associations between demographic and work-related factors with two body sites musculoskeletal symptoms.

**Variables**		OR	95%CI		P for trend
**Gender**	Female	**1.760**	**1.377**	**2.263**	-
**Physical exercise**	Occasionally	1.030	0.848	1.256	0.037
	2–3 times/month	0.876	0.561	1.324	
	1–2 times/week	0.829	0.567	1.186	
	More than 3 times/week	**0.581**	**0.342**	**0.934**	
**Smoking behavior**	Occasionally	1.272	0.924	1.736	0.001
	Frequently	**1.443**	**1.028**	**2.009**	
**Standing for long period at work**	Occasionally	**1.343**	**1.064**	**1.694**	0.005
	Frequently	**1.390**	**1.039**	**1.855**	
	Always	**1.721**	**1.243**	**2.375**	
**Sitting for long period at work**	Occasionally	1.024	0.786	1.335	0.001
	Frequently	**1.406**	**1.067**	**1.857**	
	Always	**1.502**	**1.082**	**2.083**	
**Squatting or kneeling for long period at work**	Occasionally	**0.730**	**0.570**	**0.929**	0.301
	Frequently	0.918	0.513	1.540	
	Always	1.499	0.777	2.709	
**Work in an uncomfortable position**	Occasionally	**1.389**	**1.144**	**1.686**	0.008
	Frequently	1.358	0.962	1.883	

	Always	1.286	0.755	2.096	
**Performing the repetitive operations per minute**	Occasionally	1.165	0.893	1.523	0.022
	Frequently	1.075	0.831	1.395	
	Always	**1.429**	**1.082**	**1.890**	
**Carrying heavy objects >5 Kg**	Occasionally	1.083	0.881	1.331	0.445
	Frequently	0.802	0.586	1.088	
	Always	**1.583**	**1.053**	**2.343**	

Table S3 Multivariate logistic regression analysis describing the associations between demographic and work-related factors with more than two body sites musculoskeletal symptoms.

**Variables**		OR	95%CI		P for trend
**Gender**	Female	**1.565**	**1.305**	**1.881**	-
**Age (years old)**	26–35	0.810	0.653	1.006	0.322
	>35	**0.765**	**0.594**	**0.987**	
**Monthly income (RMB)**	3001-5000	**0.749**	**0.641**	**0.877**	<0.001
	>5000	**0.772**	**0.629**	**0.946**	
**Physical exercise**	Occasionally	0.947	0.818	1.098	0.001
	2–3 times/month	1.017	0.743	1.379	
	1–2 times/week	0.640	0.478	0.849	
	More than 3 times/week	**0.688**	**0.480**	**0.969**	
**Job tenure (years)**	3–5	0.918	0.775	1.085	<0.001
	>5	**1.382**	**1.174**	**1.628**	
**Standing for long period at work**	Occasionally	0.967	0.807	1.157	0.001
	Frequently	**1.270**	**1.019**	**1.584**	
	Always	**1.652**	**1.293**	**2.113**	
**Sitting for long period at work**	Occasionally	0.900	0.736	1.101	<0.001
	Frequently	**1.670**	**1.350**	**2.069**	
	Always	**2.069**	**1.618**	**2.648**	
**Squatting or kneeling for long period at work**	Occasionally	0.992	0.836	1.176	0.12

	Frequently	**0.632**	**0.418**	**0.937**	
	Always	1.070	0.647	1.740	
**Work in an uncomfortable position**	Occasionally	**1.722**	**1.489**	**1.991**	<0.001
	Frequently	**3.085**	**2.462**	**3.863**	
	Always	**3.014**	**2.152**	**4.220**	
**Performing the repetitive operations per minute**	Occasionally	**1.302**	**1.040**	**1.635**	<0.001
	Frequently	**1.567**	**1.268**	**1.942**	
	Always	**2.328**	**1.865**	**2.915**	
**Carrying heavy objects >5 Kg**	Occasionally	**1.294**	**1.105**	**1.517**	0.001
	Frequently	**1.458**	**1.183**	**1.795**	
	Always	1.232	0.911	1.659	
**Exerting great force with arms or hands**	Occasionally	1.037	0.846	1.271	<0.001
	Frequently	**1.353**	**1.106**	**1.657**	
	Always	**1.406**	**1.119**	**1.766**	

Figure S1. Multivariate logistic regression analysis describing the associations between demographic and work-related factors with more than two body sites musculoskeletal symptoms.

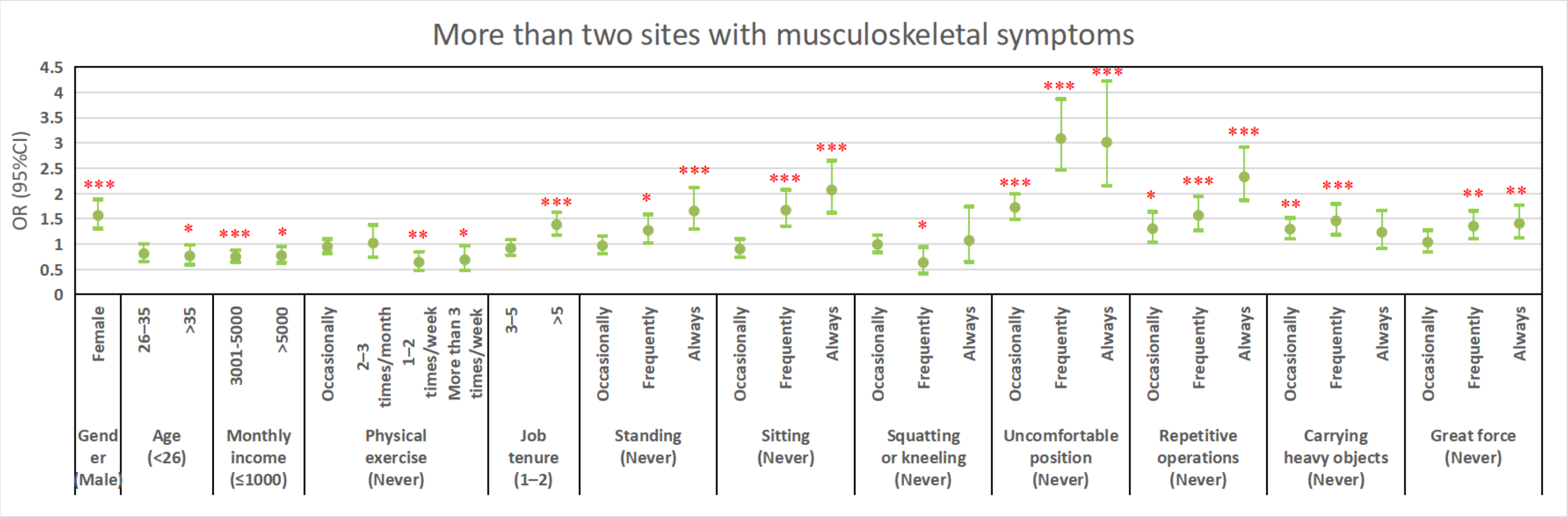

Notes: *, P<0.05; **, P<0.01; ***, P<0.001.

Figure S2. Multivariate logistic regression analysis describing the associations between demographic and work-related factors of the neck and shoulder musculoskeletal symptoms.

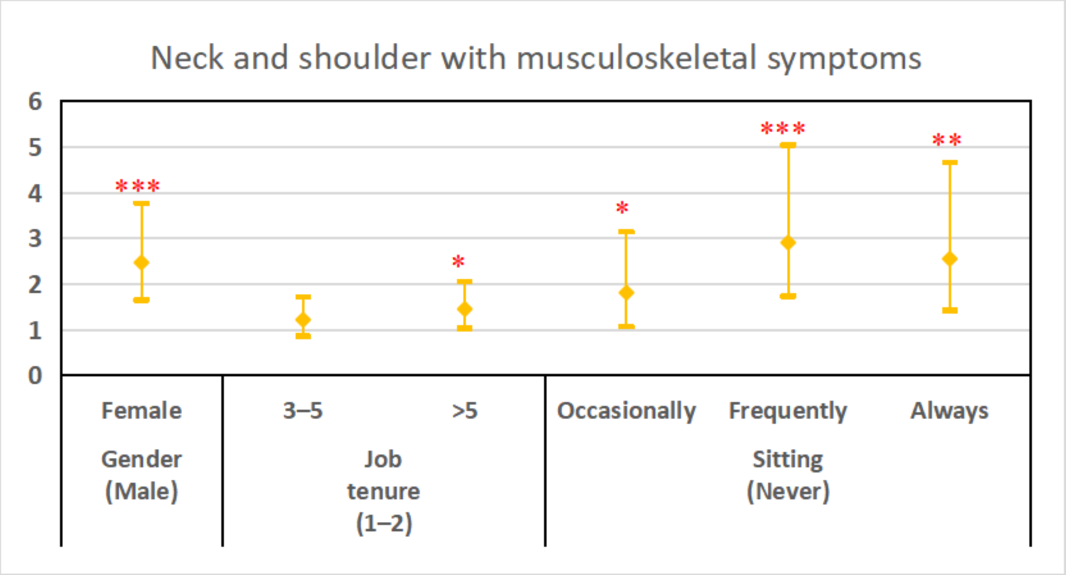

Supplement: Supplementary file 1 [file ijerph-19-13315-s001.zip › ijerph-1932994-supplementary.pdf]
